# 

**Published:** 1900-12-08

**Authors:** 


### NOTICE TO CORRESPONDENTS.

All MSS., Letters, Books for Review, and other matters intended for the Editor should be addressed The Editor, "The Hospital," The Hospital Building, 28 & 29 Southampton Street, Strand, W.C.

The Editor cannot undertake to return rejected MSS., even when accompanied by stamped directed envelope.

**Notice to Advertisers and Subscribers.**

All Advertisements, Orders for Copies of The Hospital, and Business Communications should be addressed to **THE MANAGER (Not to the Editor)**, The Hospital, 28 & 29 Southampton Street, Strand, London, W.C.

The Cost of Subscription, post free, is as follows:—Per week, 2½d.; per quarter, 2s. 8d.; per half-year, 5s. 3d.; per annum, 10s. 6d. Foreign:—Per annum, 15s. 2d., payable, in all cases, in advance.

**NOTICE.—Vol. XXVIII. of "The Hospital" (April to September, 1900), in handsome cloth binding, is now on sale at the Office of this Journal, price, 7s. 6d. Cases for binding the Numbers contained in this Volume 1s. 6d. Index to Vol. XXVIII., 2½d., post free.**

**The Monthly Part for December is now ready. Price 6d., by post 8d.**

### BOOKS RECEIVED.

H. K. Lewis.

"What to do in Cases of Poisoning." By William Murrell, M.D., F.R.C.P.

Oppenheimer, Son and Co.

"Ephemeris Pharmacologica."

The Sanitary Publishing Company.

"The Food Inspector's Handbook." By Francis Vacher.

Rebman, Limited.

"Certification and Classification of 'Diarrhœa' Deaths." Reprinted from "Public Health."

Longmans, Green and Co.

"On the Use of Massage and Early Passive Movements in Recent Fractures, and the Treatment of Internal Derangements of the Knee-joint." By William H. Bennett, F.R.C.S.

### SCRAPS AND GLEANINGS.

Sir Frederick Wills has given £1,000 to the committee of the new Royal Boscombe and West Hants Hospital to clear the Wills ward from debt.

The annual report of the Bristol Cottage Hospital states that during the year 1,487 cases have been treated, 98 of which were maternity cases, 35 cases of diphtheria, and 23 cases of scarlet fever.

The number of accidents treated during the year at the Ancoats Hospital was 8,939, being 831 in excess of the previous year. The number of in-patients apart from accident cases was 1,402.

The completed portion of the New Royal Boscombe Hospital which has just been opened has cost £8,000, exclusive of the land, furniture, and extras towards which £5,800 has been subscribed or promised.

The amount handed over to the local hospitals by the Shrewsbury Hospital Saturday Fund during the last six years averages £196 per annum. The amount available for distribution this year was £231 odd.

The committee of the Ancoats Hospital have in contemplation a scheme to pull down the west wing and erect upon the site a home for the nurses and a nursery for the children. They are also going to erect a convalescent home on a site to be chosen, for which purpose they have received a gift of £10,000.

The hospital for the outdoor treatment of consumptives which is to be erected near Bradford-on-Avon is intended for the use of patients from Wiltshire, Somersetshire, and Gloucestershire. The building will contain 50 or 60 beds: it will be under the care of the Society for the Prevention of Consumption.

The Sheffield Town Trustees have given £1,500 towards the extensions of the Sheffield Children's Hospital. These alterations and additions comprise a new out-patients' department and a new administrative department, including accommodation for the house surgeon, matron, and nurses. It is calculated that the scheme will cost £10,000.

The Hovis Company have presented Nazareth House, Dr. Barnardo's Homes, and other similar institutions with sufficient Hovis loaves to enable the inmates to have a Hovis tea. The loaves so presented were those submitted for inspection as to quality in connection with a competition among the Hovis agents in the south-east of England.

The financial statement presented to the recent annual meeting of the Liverpool Hospital Saturday and Sunday Committee showed the results of this year's collection to be £13,879, of which sum the church collections produced £6,058, and the Saturday boxes £7,103, the balance brought forward from last year being £668. The grants made this year by the Saturday and Sunday Fund Committee amounted to £12,805.

Mr. John Lawson Johnston, the inventor of Bovril, who has just died, was the possessor of the Royal Humane Society's Gold Medal, conferred upon him for saving life on several occasions. He was also a Fellow of the Red Cross Society of France, which distinction was conferred upon him by the French Government in recognition of his services as special commissioner for that country in connection with the investigations concerning food concentration and preservation.

### THE STUDENT OF MEDICINE.

### APPOINTMENTS.

**C. W. Edwards**, F.R.C.S.Edin., appointed Certifying Factory Surgeon for the Wincanton District of Somersetshire. **Andrew Fullerton**, M.D., &c., appointed Surgical Registrar to the Royal Victoria Hospital, Belfast. **Henry R. Kenwood** M.B., C.M.Edin., appointed Medical Officer of Health for the Borough of Stoke Newington. **Lawrie Hugh McGavin**, F.R.C.S.Eng., appointed Assistant-Surgeon to the North-West London Hospital. **S. H. Merryweather**, L.R.C.P., L.R.C.S.Edin., appointed Medical Officer for the Pelhams Land District of the Boston Union. **Z. B. Mudge**, L.R.C.P.Lond., M.R.C.S.Eng., appointed Certifying Factory Surgeon for the National Explosives Company, Upton Towans, Hayle. **W. Jones Richards**, M.R.C.S.Eng., L.R.C.P.Lond., appointed Fourth Assistant Medical Officer to the Middlesex County Asylum, Tooting, S.W. **J. C. R. Richardson**, M.A.Camb., M.B., B.C., appointed Medical Officer of Health to the Saxmundham Urban District **G. W. Roll**, M.B., B.C.Cantab., F.R.C.S.Eng., Assistant-Surgeon to the Royal Westminster Ophthalmic Hospital. **J. W. Smith**, M.B., F.R.C.S., appointed Honorary Assistant-Surgeon and Superintendent to the Cancer Pavilion and Home, Manchester. **W. B. Stanford**, L.R.C.P., M.R.C.S., appointed Medical Officer for the Sixth District of the Hollingbourn Union. **William Starkey**, M.B., B.Ch.R.U.I., appointed Assistant Medical Officer to the Down District Asylum, Downpatrick. **H. C. Woodhouse**, M.B., Ch.B.Vict., appointed Medical Officer for the Dawley District of the Madeley Union.

ST. THOMAS'S HOSPITAL HOUSE APPOINTMENTS.—**H. M. Harwood**, M.A., M.B., B.C.Cantab. (Extension), **R. B. Kinloch**, L.R.C.P., M.R.C.S. (Extension), **C. F. Selous**, L.R.C.P., M.R.C.S., **H. H. R. Clarke**, L.R.C.P., M.R.C.S., House Physicians. **Z. Mennell**, L.R.C.P., M.R.C.S., **F. B. Skerrett**, B.Sc.Lond., L.R.C.P., M.R.C.S., Assistant House Physicians. **A. E. Martin**, M.A., M.B., B.C.Cantab., L.R.C.P., M.R.C.S., **C. L. Hawkins**, M.A., M.B., B.C.Cantab., L.R.C.P., M.R.C.S., **J. F. Cunningham**, L.R.C.P., M.R.C.S., **Y. Takaki**, L.R.C.P., M.R.C.S. (Extensions), House Surgeons. **T. H. Edwards**, L.R.C.P., M.R.C.S., **B. S. Wills**, L.R.C.P., M.R.C.S., **T. S. Taylor**, M.A., M.B., B.C.Cantab., **T. Burfield**, M.A., M.B., B.C.Cantab., L.R.C.P., M.R.C.S. (Extensions), Assistant House Surgeons. **H. R. Beale**, L.R.C.P., M.R.C.S. (Senior), **E. W. Hedley**, M.A., M.B., B.C.Cantab. (Junior), Obstetric House Physicians. **C. A. R. Nitch**, M.B.Lond., L.R.C.P., M.R.C.S., Clinical Assistant in the Throat Department. **L. S. Dudgeon**, L.R.C.P., M.R.C.S. (Extension), Clinical Assistant in the Electrical Department.

